# Real‐world oncological and toxicity outcomes with the Moscow strain of intravesical BCG for non‐muscle invasive bladder cancer—Implications for global shortage

**DOI:** 10.1002/bco2.70034

**Published:** 2025-06-10

**Authors:** Amandeep Arora, Sugam Godse, Mahendra Pal, Ankit Misra, Ravi Teja Sepuri, Naveen Thimiri Mallikarjun, Ajit Gujela, Sachin Patel, Anuj Sharma, Santosh Menon, Ganesh Bakshi, Gagan Prakash

**Affiliations:** ^1^ Division of Uro‐Oncology, Department of Surgical Oncology Advanced Centre for Treatment, Research and Education in Cancer (ACTREC), Tata Memorial Centre, Homi Bhabha National Institute Mumbai India; ^2^ Division of Uro‐Oncology, Department of Surgical Oncology Tata Memorial Hospital, Homi Bhabha National Institute Mumbai India; ^3^ Department of Urology Kurnool Medical College Kurnool India; ^4^ Division of Uro‐Oncology, Department of Surgical Oncology Homi Bhabha Cancer Hospital and Research Centre New Chandigarh India

**Keywords:** adequate BCG, bladder cancer, intravesical BCG, Moscow strain, non‐muscle invasive

## Abstract

**Objective:**

This study aims to determine the oncological effectiveness and adverse‐effect profile of the Moscow strain of intravesical bacille Calmette–Guérin (BCG) for intermediate and high‐risk non‐muscle invasive bladder cancer (NMIBC).

**Materials and methods:**

We performed a retrospective search of consecutive intermediate and high‐risk non‐muscle invasive bladder cancer patients who were started on intravesical BCG at 80 mg dose from January 2020 to December 2021. Data were collected for oncological outcomes and adverse effects of BCG. High‐grade recurrence‐free survival (HGRFS) was defined as any relapse of high‐grade (HG) urothelial cancer or carcinoma in situ (CIS). The primary outcome was to determine the HGRFS for those with originally HG disease. The RFS and HGRFS were calculated for the entire cohort, and also stratified by whether the patients had received adequate BCG or not.

**Results:**

We identified 166 patients during the study period, of which 79.6% had HG disease. There were 25 recurrences (15.1%) in the entire cohort over a median follow‐up of 29 months. The RFS for the entire cohort at 12 and 24 months was 89.8% and 86.7%, respectively. For those with baseline HG disease, the 12‐ and 24‐month HG‐RFS was 90.9% and 87.1%. For the overall cohort, those who had received adequate BCG (*n* = 130, 78.3%) had a 12‐ and 24‐month RFS of 96.9% and 95.4%, which was significantly higher than those who were not able to receive adequate BCG (*n* = 31, 18.6%) (12‐ and 24‐month RFS of 74.2% and 64.5%), *p* < 0.001. Around 10% patients dropped out at each sequential maintenance phase, either because of BCG intolerance or because of failure to comply with the BCG instillation schedule. Severe side effects led to BCG discontinuation in 38.5% patients.

**Conclusion:**

The Moscow strain BCG at 80 mg dose has excellent oncological outcomes, especially in patients who can take adequate BCG instillations, but BCG intolerance is a problem in a significant proportion of patients.

## 1 INTRODUCTION

Intravesical Bacillus Calmette–Guerin (BCG) therapy has long been the cornerstone of treatment for non‐muscle invasive bladder cancer (NMIBC).^1^ BCG induces a local immune response in the bladder urothelium and leads to a reduction in the risk of disease recurrence and progression.^2^ Patients who are able to take BCG at the optimal dose and for the optimal duration, including the maintenance phase, show the best results.^3^ However, BCG‐induced side effects may preclude a proportion of patients (ranging from 10% to 30%) from taking BCG at the full dose, and/or, for the complete duration of 1 year (for intermediate‐risk NMIBC) or 3 years (for high‐risk NMIBC).^4^, ^5^

Various strains of BCG are used across the world. While some studies have shown superiority of the Connaught strain over the Tice strain, systematic reviews and meta‐analyses have failed to demonstrate any superiority of one strain over the other.^1^, ^6^, ^7^, ^8^, ^9^ In India, the Danish 1331 strain was available until 2016 and was used at the 120 mg dose.^1^ This strain was then discontinued and replaced by the Moscow strain which is currently the only available strain in India. With the strain change, however, the dose did not change and BCG with the Moscow strain was still widely used at 120 mg.

There is a single report from India regarding the use of this strain at 120 mg dose, and the authors report that two thirds of patients had moderate to severe adverse effects with more than 40% requiring dose reduction or discontinuation.^10^ Another report on this strain noted significantly lower side effects with the 80 mg dose.^11^ A recently published report from Switzerland was the first to report the use of this strain in the European population.^12^ To the best of our knowledge, these also happen to be the only reports in literature about the clinical use of the Moscow strain.

Following an initial experience of a high incidence of adverse effects with 120 mg dose, we shifted our practice to using the Moscow strain at the 80 mg dose. In view of the global BCG shortage, the EVER trial in Canada is currently evaluating the safety and efficacy of the Moscow strain at the 80 mg dose, in comparison to the routinely used, but not easily available, TICE strain.^13^

In this study, we sought to determine the oncological effectiveness and adverse effect profile of the Moscow strain of intravesical BCG when used at the 80 mg dose.

## 2 METHODS

### 2.1 Study design

We performed a retrospective search of our prospectively maintained institutional database of patients started on intravesical BCG therapy for NMIBC. We changed to the 80 mg dose in 2020, and we wanted to check for 2‐year oncological outcomes. Hence, we included all patients who started on BCG therapy from January 2020 to December 2021 and collected follow‐up data. The study included all consecutive intermediate and high‐risk urothelial NMIBC patients who were BCG naïve. The patients were excluded if they had variant histology, prior exposure to intravesical BCG or chemotherapy, NMIBC as a recurrence in patients treated with radiation therapy for muscle‐invasive bladder cancer or if they had a concurrent upper tract or prostatic urethral tumour. The study was approved by the institutional ethics committee (TMH/IEC III/900805).

The European Association of Urology (EAU) risk stratification was used to categorise patients into intermediate and high risk.^14^ The patients who had their transurethral resection of bladder tumour (TURBT) at another centre and had T1 high‐grade (HG) disease, underwent a re‐TURBT at our centre before proceeding to BCG therapy. For patients undergoing the initial TURBT at our centre, the re‐TURBT was performed only for patients with multiple and/or large T1 tumours. All TURBTs at our centre were performed either by fellowship‐trained uro‐oncologists or by fellows under the supervision of seniors. All patients underwent a check cystoscopy, urine cytology and a urine culture testing every 3 months in the first 2 years and 6‐monthly in the third year. Enhanced cystoscopy techniques like blue‐light cystoscopy or narrow band imaging were not used. Upper tract imaging was performed annually.

Data were collected for demographic variables, tumour characteristics, histopathology, the oncological outcomes and the side effect profile with BCG. Adverse effects were graded according to Common Terminology Criteria for Adverse Events version 5.0 (CTCAE v5.0) for bladder spasms, cystitis and dysuria. We also checked for discontinuation for reasons besides recurrence, including toxicity and failure to comply with instillation schedule, over the course of the BCG therapy. Only those patients who had not had a recurrence by the time they discontinued BCG were included for this analysis.

### 2.2 The intravesical therapy

The patients were started on intravesical BCG therapy with the Moscow strain at 80 mg dose about 3–4 weeks after the TURBT (or the re‐TURBT). The Moscow strain BCG is available as a 40‐mg vial containing 1–19.2 × 10^8^ colony forming units (CFUs). Two such vials, containing 2–38.4 × 10^8^ CFU, were reconstituted in 50 cc of normal saline for each instillation. The SWOG 8507 instillation schedule was followed, which consisted of an induction phase of six weekly instillations followed by a maintenance phase of three weekly instillations at 3, 6 and 12 months for intermediate‐risk patients and continued at 18, 24, 30 and 36 months for high‐risk patients.^15^ It is our practice to give two doses of ofloxacin 400 mg after each dose, one 6 h after instillation and another after 12 h.^16^

### 2.3 Definitions

Adequate BCG therapy was defined as completion of at least 5 out of 6 doses of the induction course plus at least two out of three doses of the first phase of maintenance therapy within a 6‐month period.^17^

Recurrence was defined as any relapse, HG or low‐grade (LG), within the bladder. An upper tract recurrence in the absence of a bladder recurrence was not considered a recurrence. Recurrence‐free survival (RFS) was defined as the time period between the start of the induction phase of BCG therapy and the event of recurrence. Accordingly, high‐grade RFS (HGRFS) was defined as any relapse of HG urothelial cancer (any T stage) or carcinoma in situ (CIS). The primary outcome of the study was to determine the HGRFS for those with originally HG disease. The RFS and HGRFS were calculated for the entire cohort, and also stratified by whether the patients had received adequate BCG or not.

### 2.4 Statistical analyses

Descriptive data were expressed as means, medians or proportions. Survival curves were generated using the Kaplan–Meier method and compared using the log‐rank test. All statistical analyses were performed using IBM SPSS Statistics for Windows, version 24 (IBM Corp., Armonk, N.Y., USA).

## 3 RESULTS

### 3.1 Patient characteristics

Figure S1 summarises the inclusion of patients for this study. The clinico‐pathological characteristics of the 166 patients who were started on BCG therapy at the 80 mg dose are depicted in Table 1. Around 65% of the patients had T1 disease. As per the EAU risk stratification, 68.1% of the patients belonged to the high‐risk category while the remaining 31.9% had intermediate‐risk disease.

**TABLE 1:** Clinico‐pathological characteristics of patients started on intravesical BCG therapy with the Moscow strain at 80 mg dose during the study period (*n* = 166).

Variables	Total *n* = 166
Age in years, median (range)	67 (36–74)
Gender, *n* (%)	
Male	139 (83.7%)
Female	27 (16.3%)
Re TURBT, *n* (%)	93 (56.2%)
*T* stage, *n* (%)	
Ta	59 (35.5%)
T1	107 (64.5%)
Patients with CIS	17 (10.2%)
Grade of tumour, *n* (%)	
Low grade	34 (20.4%)
High grade	132 (79.6%)
EAU risk category, *n* (%)	
*Intermediate risk*	53 (31.9%)
Ta low grade	34 (20.4%)
Ta high grade	19 (11.5%)
*High risk*	113 (68.1%)
Ta high grade	6 (3.6%)
T1 high grade	107 (64.5%)

*Note*: BCG = Bacillus Calmette–Guerin; TURBT = Trans‐urethral resection of bladder tumour; CIS = Carcinoma in situ; EAU = European Association of Urology.

### 3.2 Discontinuation during induction and maintenance BCG therapy **(**Figure 1 **)**

**FIGURE 1:**
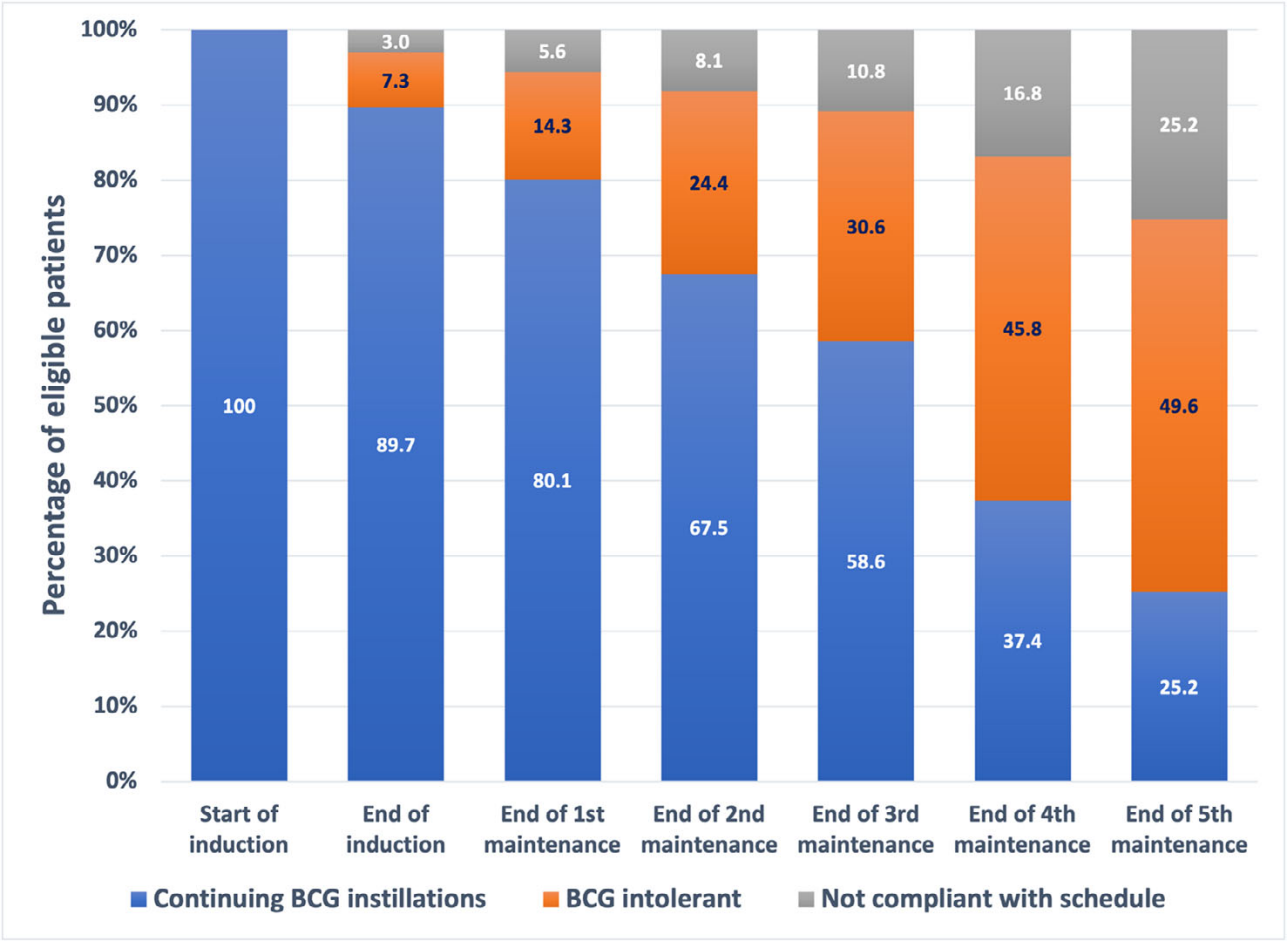
Course of patients over the sequential instillation phases. Only those patients were included for analysis who had not had a recurrence.

Of the 166 patients, 148 (88.5%) completed all six instillations of the induction therapy. Of the 18 patients who did not complete the induction phase, 12 (8.1%) had BCG intolerance. The number of patients who could not tolerate more than 1, 2, 3, 4 and 5 doses were 2, 3, 2, 3 and 2, respectively. Five other patients did not comply with the instillation schedule despite no toxicity, and one more patient had haematuria while on the induction phase and was detected with a NMIBC recurrence.

Of the 148 patients who completed the induction phase, seven (4.8%) did not proceed to the maintenance phase because of persistent urinary symptoms despite the time gap (median 8 weeks [interquartile range 6–11]) between the completion of the induction phase and the start of the first maintenance phase. Three other patients were detected with a recurrence on the cystoscopy performed prior to the start of the first maintenance phase (two were non‐muscle invasive and one muscle invasive), while one other patient had a locally advanced right ureter recurrence for which he underwent a nephroureterectomy followed by adjuvant chemotherapy. Four other patients defaulted and did not comply with the maintenance schedule despite no intolerance. The remaining 133 patients started the first maintenance phase. The percentage of eligible patients (those without a recurrence by that time) completing/discontinuing the sequential maintenance phases is depicted in Figure 1. Around 10% patients dropped out at each maintenance phase, either because of BCG intolerance or because of failure to comply with the BCG instillation schedule. A sharp fall of 22% was seen between the third and fourth maintenance phases.

### 3.3 Oncological outcomes

**FIGURE 2:**
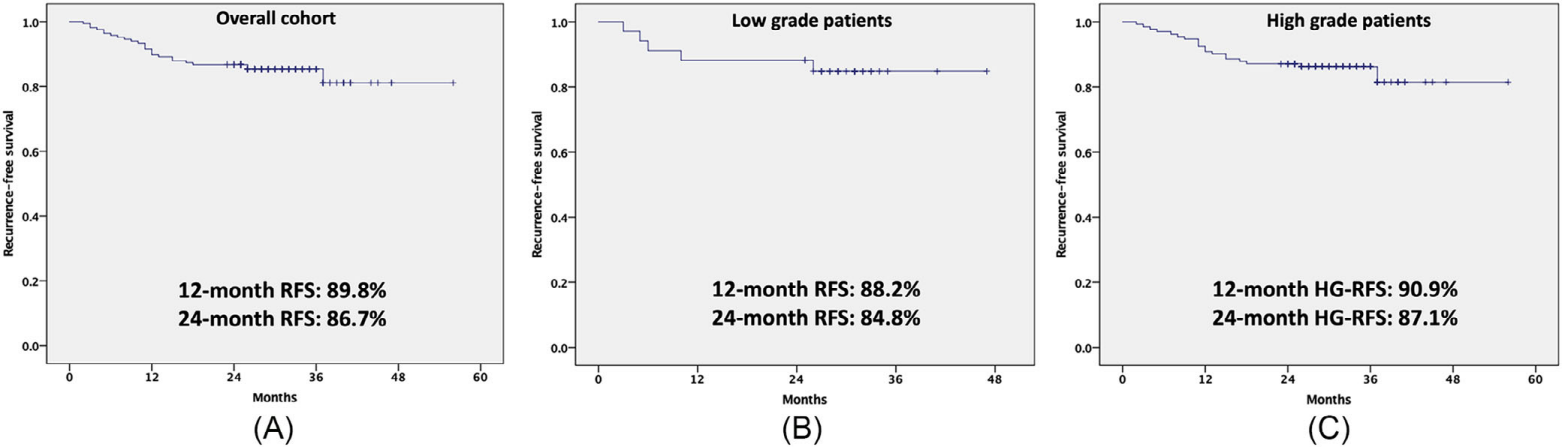
Showing the recurrence‐free survival for (A) the entire cohort of 166 patients and (B) the cohort of 32 low‐grade patients; and the high‐grade recurrence‐free survival for (C) the 134 high‐grade patients.

There were 25 recurrences (15.1%) in the entire cohort over a median follow‐up of 29 months. The RFS for the entire cohort at 12 months and 24 months was 89.8% and 86.7%, respectively (Figure 2). There were five recurrences in the 34 patients with low‐grade disease, with 12‐ and 24‐month RFS of 88.2% and 84.8%, respectively. Four of these were low‐grade recurrences and did not lead to discontinuation of further BCG instillation. One patient had a HG non‐muscle invasive recurrence. Of the 132 patients with HG disease, 20 (15.1%) had a recurrence of which only one was a low‐grade recurrence. For this cohort of baseline HG disease patients, the 12‐ and 24‐month HGRFS was 90.9% and 87.1%. All but two recurrences occurred within the first 18 months (Figure 2).

**FIGURE 3:**
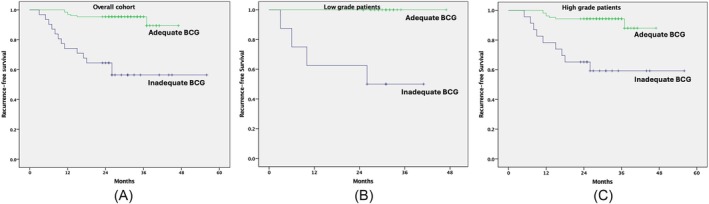
Showing the recurrence‐free survival stratified by whether adequate BCG was received or not for (A) the entire cohort of 166 patients and (B) the cohort of 32 low‐grade patients; and the high‐grade recurrence‐free survival for (C) the 134 high‐grade patients (red line = adequate BCG; blue line = inadequate BCG). BCG, Bacillus Calmette–Guerin.

For the overall cohort, those who had received adequate BCG (*n* = 130, 78.3%) had a 12‐ and 24‐month RFS of 96.9% and 95.4%, which was significantly higher than those who were not able to receive adequate BCG (*n* = 31, 18.6%) (12‐ and 24‐month RFS of 74.2% and 64.5%), *p* < 0.001. A similar difference was noted when low‐grade and HG patients were stratified for RFS as per receipt of adequate BCG (Figure 3). For this ‘adequate BCG’ analysis, one patient who had a recurrence while on induction therapy, another patient who had a locally advanced upper tract recurrence after induction therapy, and an additional three patients who recurred before starting the first maintenance phase were excluded, as they technically could not have gone on to receive adequate instillations despite no intolerance. Of the 25 total recurrences, eight were muscle invasive.

### 3.4 Adverse‐effect profile

The adverse effects (bladder spasms, cystitis and dysuria), as captured by the CTCAEv5.0, and consequent dose reduction or BCG discontinuation is summarised in Table 2. Twelve of the 166 (7.2%) patients who started the BCG therapy could not complete the induction phase because of BCG intolerance. Another seven patients did not start the maintenance phase because of persistent CTCAEv5.0 grade 3 toxicity. For severe side effects, 13 (7.8%) required a short course of oral prednisolone. One patient had to be started on anti‐tubercular therapy for histologically proven BCG‐induced bladder tuberculosis, while another patient needed a cystectomy for a dysfunctional small‐capacity bladder with upper tract changes.

**TABLE 2:** Adverse effects related to BCG instillation and its impact on further treatment.

No adverse effects at 80 mg	42 (25.3%)
CTCAE grades 1 and 2 with 80 mg but no dose reduction	46 (27.7%)
CTCAE grades 1 and 2 requiring dose reduction to 40 mg	14 (8.4%)
CTCAE grades 2 and 3 leading to BCG discontinuation (BCG intolerance)	64 (38.5%)
Intolerance during induction	12
Intolerance prior to first maintenance	7
Intolerance after first maintenance	17
Intolerance after second maintenance	9
Intolerance after third maintenance	12
Intolerance after fourth maintenance	5
Intolerance after fifth maintenance	2

*Note*: BCG = Bacillus Calmette–Guerin; CTCAE, Common Terminology Criteria for Adverse Events.

## 4 DISCUSSION

With close to half a century of using intravesical BCG to treat bladder cancer, there have been numerous reports of how different strains have performed in various parts of the world. The Moscow strain, also known as the Russia strain, has only been available over the last 6–8 years, and thus, only a handful of reports exist regarding its use.^10^, ^11^, ^12^, ^18^ It was made available in India around 2016 and is now being evaluated for use in the Western population, considering the shortage of the TICE and Connaught strains.^12^ We present here the largest experience with intravesical Moscow strain BCG for NMIBC.

We noted excellent oncological outcomes with the Moscow strain, with 1‐year RFS close to 90% and 2‐year RFS more than 85%. This was seen for the overall cohort, and also when evaluated separately for low‐grade and HG patients. There have been three prior reports with the Moscow strain in the Indian population. The first of them by Yuvaraja et al. had in fact compared the Danish 1331 strain (available in India prior to 2016) with the Moscow strain and had found no difference in the recurrence rates.^10^ The 3‐year RFS with the Moscow strain was 72.9%. The same in our cohort was considerably higher at 85.4%. The lower RFS in their study can possibly be explained by the fact that 67% of their patients experienced moderate to severe side effects, leading to BCG discontinuation in 43% patients. While the details about what stage during the course of BCG therapy these discontinuations took place are not available in the paper, the inability of the patients to take enough BCG instillations could have led to a higher recurrence rate. It is interesting to note that patients in that series had received Moscow BCG at a higher dose of 120 mg which could have led to a higher incidence of side effects. We have earlier highlighted the plausible explanation for this higher toxicity related to this strain and emphasised the need to tailor the dose of BCG based on toxicity.^1^ Another report from India, which was led by the manufacturer of Moscow BCG in India, also showed a 3‐year RFS of 84.3% with 80 mg dose.^11^ The third report from India was mainly focussed on BCG‐related complications and mentioned that 15% patients failed with BCG (without a mention of the timeframe); this also seems comparable to our finding.^18^ However, report does not mention the dose at which BCG was used. Overall, Moscow strain BCG has shown excellent RFS across all reports in the Indian population. Also, the fact that almost all our recurrences occurred during the first 18 months of the BCG therapy should possibly help us re‐assure our patients who have made it to around 24 months without a recurrence.

A more recently published report of the use of Moscow strain in the Swiss population yielded average outcomes with 1‐, 3‐ and 5‐year RFS of 76%, 65% and 61%, respectively.^12^ To the best of our knowledge, this is the only published data with this strain in the western population. This study used the Moscow strain at 120 mg dose, but early BCG discontinuation leading to inadequate BCG therapy was seen in only 4% patients, signifying that the 120 mg dose was well tolerated by this population. The study continued maintenance BCG instillations for only a year (instead of 3 years for high‐risk patients); and the authors postulated that this might explain their higher recurrence rate. However, the 76% one‐year RFS suggests that patients had recurrences even when they were receiving BCG during the first year and not necessarily after they stopped the maintenance phase after 1 year. In contrast to the western countries, the Indian population is heavily immunised with BCG. The phenomenon of ‘trained immunity’ may be applicable here; wherein a primary stimulus (here, BCG vaccination in childhood) trains the immune system to lodge a heightened response to a secondary stimulus (here, intravesical BCG instillation).^19^, ^20^ It remains to be explored whether this was the reason for the reduced oncological effectiveness, and also lesser toxicity, of the Moscow strain BCG in a non‐immunised population.

With regards to adverse effects, we noted a sustained drop‐out rate of around 10% with every sequential maintenance phase. The majority of this was attributed to BCG intolerance, with a small proportion of patients also dropping out because of failure to comply with the maintenance schedule. The biggest fall of >20% was seen after around 1 year of BCG therapy when patients had completed three maintenance phases. Our side‐effect profile can be deemed substantial with about a third of the patients experiencing moderate to severe symptoms. This is very similar to the toxicity profile reported in another Indian report.^18^ On the contrary, the study by Yuvaraja et al. reported much higher toxicity with moderate to severe symptoms in 67% patients, possibly explained by the use of 120 mg dose as stated earlier. Similarly, the need for anti‐tubercular drugs and cystectomy for severe symptoms was rare in our patients compared to 20% and 11% in this other study.^10^ The other study by the manufacturers of Moscow BCG in India noted that patients had ‘only mild symptoms which subsided within 2–3 days with analgesics, antibiotics and fluid therapy’. They, however, found a significantly higher number of adverse events in patients receiving 120 mg dose compared to those with 80 mg.^11^ The study in the Swiss population, likewise, noted Grade 3 complications in only 8.9% patients.^12^

We noted that patients who were able to receive adequate BCG instillations did strikingly better than those who could not. In fact, none of the low‐grade patients who received adequate BCG had a recurrence. For the HG patients, only one had a recurrence during the first year of therapy. On the contrary, the inadequate BCG patients kept having recurrences all along the first 2 years after starting BCG therapy. This finding is in sync with other reports and validates the International Bladder Consensus Group (IBCG) definition of adequate BCG.^17^, ^21^, ^22^, ^23^ This impact of adequate BCG on RFS has not been reported in the other studies with the Moscow strain.

Taken together, our results indicate that the Moscow strain when adequately received is efficacious and has RFS rates at par or better than the other strains of BCG which are currently facing a shortage. This effectiveness however is accompanied by intolerance in one third of our patients and inability to receive adequate BCG in one fifth. Thus, the challenge lies in finding strategies that can enable our patients to take those adequate instillations. We have previously suggested that one strategy could be reducing the dose of the Moscow strain because a single vial of 40 mg contains 1–19.2 × 10^8^ CFU, which is actually enough as per the original data which suggested that 10^8^–10^9^ CFU per instillation is the effective BCG dose.^1^, ^24^ Also, various BCG strains have different immunogenicity, and a prior in vitro study revealed that the Moscow strain resulted in the highest inhibition of tumour cell proliferation and also the highest production of IL‐8.^25^ This can probably be attributed to the fact that the Moscow strain is a phylogenetically ‘early BCG strain’ and has been through lesser serial passages compared to some other widely used strains like TICE, Connaught and Danish 1331.^1^, ^19^ Further dose reduction can only be advocated once it is confirmed that this does not significantly lower the RFS rates. Another strategy would be to tailor each dose in the instillation schedule so as to enable an increasing proportion of patients to take adequate instillations.^26^

In contrast to the global shortage with the TICE strain, which is the predominantly used strain in the western population, the Moscow strain is widely available across India and is also being evaluated in the EVER trial in Canada. Thus, this strain could potentially become the most commonly used strain across the world in the future. The BCG shortage has also led to clinicians using alternative agents, most commonly intravesical sequential gemcitabine–docetaxel.^27^ Also, multiple other agents and device assisted therapies are currently being evaluated. BCG, however, is still the gold standard, and the Moscow strain has set the bar very high for these newer therapies with its excellent oncological effectiveness. Our efforts should be directed towards optimising its dosage and/or schedule in various populations so as to enable more patients to tolerate it without compromising the RFS.

Our study is not devoid of limitations. The retrospective design is prone to inherent biases and loss of data collection. However, almost all patients had follow‐up data for at least 2 years. Primary CIS is not commonly seen in our population, and we are not sure if these oncological outcomes would also apply to populations with a higher incidence of CIS. For patients who had moderate symptoms but still chose to continue with further instillations at the same or lower dose, we could not capture whether there was a more than usual delay between instillations and whether this could impact the RFS.

In conclusion, the Moscow strain BCG at 80 mg dose has excellent oncological outcomes in the Indian population, especially in patients who can take adequate BCG instillations. However, it also causes moderate to severe symptoms in a significant proportion of patients leading to BCG intolerance. Further studies should focus on evaluating this strain in other populations worldwide and optimising its dose/schedule to make it more tolerable without compromising its effectiveness. This can potentially help solve the global BCG shortage.

## AUTHOR CONTRIBUTIONS

Study concept and design: Amandeep Arora, Gagan Prakash, Mahendra Pal and Ganesh Bakshi. Analysis of data: Amandeep Arora, Sugam Godse, Naveen Thimiri Mallikarjun, Sachin Patel and Ravi Teja Sepuri. Drafting of manuscript: Amandeep Arora, Anuj Sharma, Sugam Godse, Ankit Misra and Ajit Gujela. Critical revision of manuscript for important intellectual content: Gagan Prakash, Ganesh Bakshi and Santosh Menon. Statistical analysis: Amandeep Arora and Naveen Thimiri Mallikarjun. Study supervision: Gagan Prakash and Mahendra Pal.

## CONFLICT OF INTEREST STATEMENT

The authors declare no conflicts of interest.

## Supporting information


**Figure S1:** Flowchart for inclusion of patients for the study
