# Supplementary figures and images for: Real‐world oncological and toxicity outcomes with the Moscow strain of intravesical BCG for non‐muscle invasive bladder cancer—Implications for global shortage
Source: BJUI Compass. 2025 Jun 10;6(6):e70034. doi: 10.1002/bco2.70034 (PMC12149984; doi:10.1002/bco2.70034)

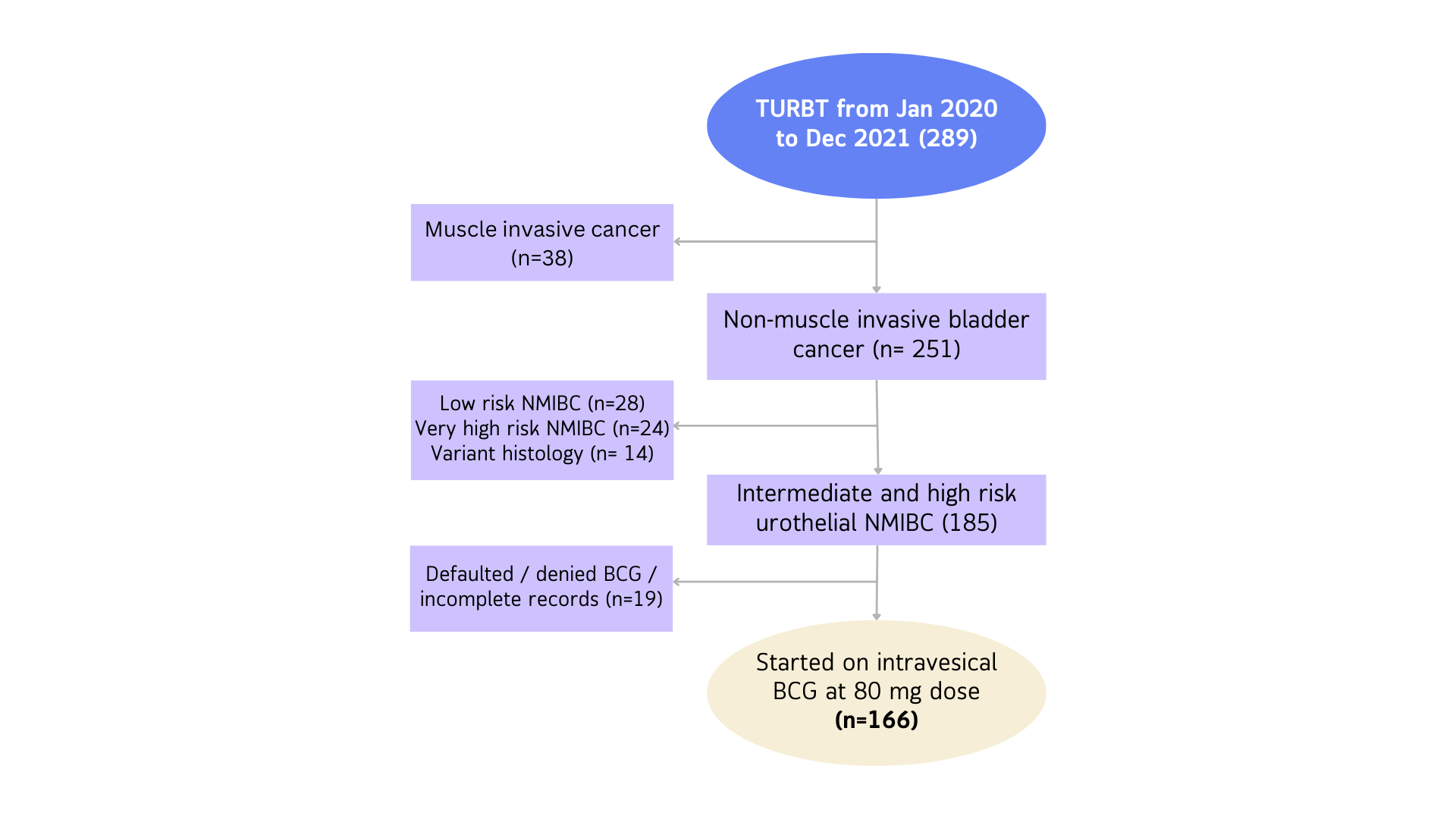

Supplement: Supplementary file 1 — Figure S1: Flowchart for inclusion of patients for the study [file BCO2-6-e70034-s001.png]
